# Social Determinants of Health: *What, How, Why,* and *Now*

**Published:** 2007-09-15

**Authors:** Marilyn Metzler

**Affiliations:** McKing Consulting, National Center for Chronic Disease Prevention and Health Promotion/DACH, Centers for Disease Control and Prevention

*Healthy People 2010 *(1), the U.S. blueprint for improving population health, captured the attention of public health practitioners across the world with its bold and explicit commitment to eliminating health disparities. The document outlines a compendium of health care system and individual behavioral change objectives to be achieved toward this end. However, one of the more insightful statements in the document is often overlooked: 

Healthy People 2010* recognizes that communities, States, and national organizations will need to take a multidisciplinary approach to achieving health equity — an approach that involves improving health, education, housing, labor, justice, transportation, agriculture, and the environment, as well as data collection itself.*

The section goes on to state that "current data collection methods make it impossible to assess accurately the health status for some populations, particularly relatively small ones." The section also acknowledges the importance of individual and community empowerment to address health disparities by promoting community safety, education, and access to health care (1).

Social determinants of health — an idea whose time has come? Not exactly. It is actually an idea that has been part of the public health story, whether in ancient or modern times, when concerned practitioners noted the need to improve poverty, sanitation, and other living conditions to improve health. The shift to biomedical and behavioral approaches to public health occurred in the 20th century with the advent of antibiotics and other medical advances, as well as with the publication of a vast psychology literature that sheds light on concepts of self-efficacy, stages of change, and other approaches to individual risk reduction. These approaches were deemed especially critical by the end of the century with the onset of the HIV/AIDS epidemic. The urgency of risk reduction as a public health strategy was further heightened by rapid, population-wide increases in obesity and chronic diseases. Knowledge of the relationship between underlying conditions and individual choice, though not well understood scientifically earlier in the century, was subsumed by research, programs, and policies focused almost exclusively on individual responsibility for health, regardless of the circumstances in which people lived. However, a rapidly growing body of literature provides mounting evidence that addressing underlying social factors is critical in the quest to eliminate health disparities (2), including significant gaps in mortality, whether measured by race (3) or by socioeconomic position (4).

The inherent beauty of the *Healthy People 2010* quotation is that one sentence encompasses key values, ideas, and strategies that underscore the growing conversation on social conditions and their impact on health. Perhaps most obvious is the acknowledged need to improve housing, education, transportation, and other resources for health — domains not currently within the public health arena. Also explicit is the need to make improvements in data collection. Although assessment is a core public health activity, data on social conditions is minimal in public health surveillance systems. Taken together, social determinants and their indicators provide an overarching framework for reconsidering many current public health activities. The *what *— social determinants and their indicators — is a key focus of several papers in this issue of *Preventing Chronic Disease* that, individually and collectively, bring thoughtful and important information to this endeavor.

From the *Healthy People 2010* statement we also understand that *how* these challenges are approached is critical if we are to successfully eliminate health disparities. We need to work across disciplines and across many levels of political and social organization. This need implies that the task of addressing social conditions for health is also about relationships, including expanding our range of partners and improving the strategies by which we approach these partnerships. Here, the importance of community empowerment is invoked, given growing recognition of communities as both settings and key actors in the quest for health. However, the common notion of empowerment as something one individual or group gives to another individual or group, as in "we need to empower communities," limits recognition of existing community assets and inhibits understanding of empowerment as both a process and a goal. Developing reciprocal, trusting, and equitable relationships with communities is a more effective strategy (5).

Which brings us to a final but perhaps the most important concept invoked in the *Healthy People 2010* statement — that of health equity. The vast majority of health disparities experienced by groups defined by race or ethnicity, socioeconomic status, or other positions of social disadvantage are avoidable. That is, they are not attributable to immutable factors such as genetics (6). These avoidable health disparities are inherently unfair (7). This is *why* it is critical to improve the social determinants of health — because, in addition to the growing evidence base, it is the fair and just thing to do.

Several papers in this issue contribute to the literature on social determinants and their indicators and are grounded in these key concepts of resources and relationships, empowerment, and equity. Braveman reminds us that poverty, one of our nation's most intransigent social problems, continues to contribute to multiple disparate health outcomes through a variety of pathways, including reduced social standing and limited access to healthy food and safe neighborhoods (8). Recent findings about health effects related to both absolute and relative income provide evidence that addressing poverty can improve health for people across the socioeconomic gradient. Understanding poverty as a problem of national significance is critical for developing large-scale policy and programmatic responses. Understanding poverty as a problem of communities and regions is equally important, a task Holt undertakes in his paper on the topography of poverty in the United States (9). Using complex spatial analytic methods, he calculates and displays county-level poverty data and identifies pockets of low and high concentrations of poverty in eloquent maps that even the most GIS (geographic information system)-impaired among us can appreciate. These displays visually represent, as Holt notes, "social landscapes that result from underlying processes" and are thus vital for the development of locally relevant strategies to address poverty and its effects on health.

The relationship between education and income is so well established that educational attainment is often used as a proxy measure of socioeconomic status when more complete measures are lacking. More education, in addition to being associated with more income, is also a predictor of better health; less education is a predictor of health disparities. Despite these strong associations, Freudenberg and Ruglis remind us that graduating from high school is rarely considered a public health priority (10). Following a summary of known health benefits of high school graduation, they examine strategies for reducing dropout rates that emphasize improving school completion by improving health; they also recommend ways to reframe high school dropout as a public health issue. Wold and Nicholas describe one community's response to the problem of school noncompletion in their paper on Los Angeles County's social indicators initiative that was developed to improve school retention rates by improving school readiness for children under 5 years of age (11). This public–private partnership among the local health department, county supervisors, child advocacy groups, and others is based on an ecological model and incorporates an understanding of the many contextual factors that can affect children's health and their chances for a successful educational experience.

The value of gathering and monitoring local data as a tool for addressing local concerns has led to the development of community indicator initiatives across the nation and around the world. An overview of the attributes and activities of many of these initiatives is provided by Flores and colleagues at Prevention Institute (12). Although these initiatives are not usually developed to explicitly address public health concerns, they are often quite sophisticated in their use of data and can provide needed evidence for addressing local issues that contribute to health disparities. If public health professionals are involved, their role is often limited in scope, yet the opportunity for public health partnerships with these groups is rich, unexplored territory. In most cases, it is reasonable to expect a time delay between efforts to improve living conditions and a resulting improvement in health. But progress can be measured and monitored along the way by assessing intermediate outcomes, an approach Hanni, Mendoza, Snider, and Winkleby discuss in their paper on organizational change (13). They propose a framework for understanding how systems and policies change as a result of community-based approaches that involve multiple, interrelated, and complex programs and partners.  

Efforts to improve the conditions for health will present many challenges, not least among them the possibility for unintended effects. In her editorial in this issue, Wilcox uses a playful image of interacting bubbles, connected to each other by dynamic processes, to explore the topic of models that seek to describe the multiple factors affecting health (14). Just as a change in one bubble creates changes in the others, even small changes in complex systems can affect the entire system. Improving disadvantaged neighborhoods by adding walking trails and full-service grocery stores can set into motion gentrification processes that displace low-income residents (15). Improving access to education can create despondency in people unable to find jobs where they can use their new skills (16). Systems modeling is one way to explore these interconnected relationships by thinking critically about plausible futures through the use of what-if scenarios (17).   

There is growing appreciation in the public health arena that addressing social determinants and their indicators is important. At the Centers for Disease Control and Prevention, this approach is included in the agency's "Healthy People in Healthy Places" goals (18), and is expressed in its most recent program announcement for Racial and Ethnic Approaches to Community Health Across the U.S. (REACH U.S.) (19), in the recently released research guide (20), and in its invitation to Sir Michael Marmot, chair of the WHO Commission on Social Determinants of Health and principal investigator of the British Whitehall studies, to present the keynote address at this year's prestigious Charles C. Shepard Science Award ceremony.

We have much to learn as we go forward. While increased understanding of social determinants of health is critical, we know enough to act *now* if we are serious about eliminating health disparities.
